# Digital teaching as an instrument for cross-location teaching networks in medical informatics: opportunities and challenges

**DOI:** 10.3205/zma001349

**Published:** 2020-11-16

**Authors:** Nils-Hendrik Benning, Martin Haag, Petra Knaup, Dagmar Krefting, Otto Rienhoff, Markus Suhr, Inga Hege, Daniel Tolks

**Affiliations:** 1 Heidelberg University Hospital, Institute of Medical Biometry and Informatics, Heidelberg, Germany; 2 HiGHmed Consortium, Working Group Teaching, Heidelberg, Germany; 3 Heilbronn University of applied sciences, GECKO Institute for Medicine, Informatics and Economics, Heilbronn, Germany; 4 German Society for Medical Informatics, Biometry and Epidemiology, WG Technology-based Teaching and Learning in Medicine, Germany; 5 Gesellschaft für Medizinische Ausbildung (Society for Medical Education), Committee on Digitization - Technology-Assisted Learning and Teaching, Erlangen, Germany; 6 University Medical Center Göttingen, Department of Medical Informatics, Göttingen, Germany; 7 University of Applied Sciences Berlin, Berlin, Germany; 8 University of Augsburg, Medical School, Medical Education Sciences, Augsburg, Germany; 9 LMU Munich, University Hospital, Institute for Medical Education, Munich, Germany; 10 Leuphana University Lüneburg, Center for Applied Health Sciences, Lüneburg, Germany

**Keywords:** education, distance, medical informatics, computer user training, teaching networks

## Abstract

The increasingly digitized healthcare system requires new skills from all those involved. In order to impart these competencies, appropriate courses must be developed at educational institutions. In view of the rapid development of new aspects of digitization, this presents a challenge; suitable teaching formats must be developed successively. The establishment of cross-location teaching networks is one way to better meet training needs and to make the necessary spectrum of educational content available. As part of the Medical Informatics Initiative, the HiGHmed consortium is establishing such a teaching network, in the field of medical informatics, which covers many topics related to the digitization of the health care system.

Various problem areas in the German education system were identified that hinder the development of the teaching network. These problem areas were prioritized firstly according to the urgency of the solution from the point of view of the HiGHmed consortium and secondly according to existing competencies in the participating societies. A workshop on the four most relevant topics was organized with experts from the German Society for Medical Informatics, Biometry and Epidemiology (GMDS), the Society for Medical Education (GMA) and the HiGHmed consortium. These are:

1. recognition of exam results from teaching modules that are offered digitally and across locations, and their integration into existing curricula;
2. recognition of digital, cross-location teaching in the teachers' teaching load;
3. nationwide uniform competencies for teachers, in order to be able to conduct digital teaching effectively and with comparable quality;
4. technical infrastructure to efficiently and securely communicate and manage the recognition of exam results between educational institutions.

For all subject areas, existing preliminary work was identified on the basis of working questions, and short- and long-term needs for action were formulated. Finally, a need for the redesign of a technologically supported syntactic and semantic interoperability of learning performance recording was identified.

## Zusammenfassung

Das zunehmend digitalisierte Gesundheitswesen erfordert neue Kompetenzen von allen Beteiligten. Um diese Kompetenzen vermitteln zu können, müssen an den Bildungsinstitutionen entsprechende Lehrveranstaltungen entwickelt werden. Dies ist angesichts der rasanten Entwicklung neuer Digitalisierungsaspekte eine Herausforderung; passende Lehrformate müssen sukzessive aufgebaut werden. Die Etablierung standortübergreifender Lehrverbünde ist eine Möglichkeit, den Ausbildungsbedarf besser zu decken und die notwendige Bandbreite an Bildungsinhalten verfügbar zu machen. Im Rahmen der Medizininformatik-Initiative wird im HiGHmed-Konsortium ein solcher Lehrverbund im Fachgebiet der Medizinischen Informatik aufgebaut, in welches viele Themen der Digitalisierung des Gesundheitswesens fallen.

Dabei wurden verschiedene Problemfelder im deutschen Bildungswesen identifiziert, welche den Aufbau des Lehrverbundes hemmen. Diese Problemfelder wurden unter Beachtung der Dringlichkeit der Lösung aus Sicht des HiGHmeducation Konsortiums und andererseits nach vorhandenen Kompetenzen in den teilnehmenden Fachgesellschaften priorisiert. Zu den vier relevantesten Themen wurde ein Workshop mit Fachleuten der deutschen Gesellschaft für Medizinische Informatik, Biometrie und Epidemiologie (GMDS), der Gesellschaft für Medizinische Ausbildung (GMA) und des HiGHmed Konsortiums ausgerichtet. Diese sind:

1. Anerkennung von Prüfungsleistungen aus Lehrmodulen, welche digital und standortübergreifend angeboten werden, und Integration in bestehende Curricula;
2. Anerkennung digitaler, standortübergreifender Lehre im Deputat der Lehrenden;
3. bundesweit einheitliche Kompetenzen für Lehrende, um digitale Lehre effektiv und mit vergleichbarer Qualität betreiben zu können;
4. technische Infrastrukturen, um die Anerkennung zwischen Bildungseinrichtungen effizient und sicher kommunizieren und verwalten zu können.

Für alle Themengebiete wurden anhand von Arbeitsfragen existierende Vorarbeiten identifiziert sowie kurz- und langfristige Handlungsbedarfe formuliert. Abschließend wurde themenübergreifend ein Bedarf für die Neugestaltung einer technologisch unterstützten syntaktischen und semantischen Interoperabilität der Erfassung von Lernleistungen festgestellt.

## Introduction

Digitalization has a far-reaching impact on everyday life in almost all areas of society. The demands on students, teachers and university employees are subject to a fundamental change process [1], [2]. According to the Hochschulforum Digitalisierung (the higher education forum for digitalization), the structural conditions at universities are favorable for innovation in digital teaching, since the high degree of autonomy provided by decentralized organization can promote innovation [2].

The establishment of cross-locational teaching networks promises to increase competence at distant institutions [3]. Both the students who are currently studying and professionals can benefit from these teaching networks. Of particular added value, compared with conventional teaching, is the fact that the learners have access to a broader catalogue of educational content. This breadth is particularly important in the digitalization of the health care system [4], [5], since the necessary competencies and structures are only gradually being built up at the various institutions [2] and are usually not fully available [6]. Learning at multiple institutions can enable the most comprehensive acquisition of digital skills for health care professionals.

While cross-location teaching has great potential, the actual implementation poses challenges for both students and teachers. The many technical, didactic, organizational and legal challenges mean that participation in courses across locations is still rare. Even the content and forms of teaching are subject to digital transformation. For example, the digital delivery of teaching materials via online learning platforms is being expanded into web-based self-study units and fully digitalized online courses [7]. The flexibility inherent in digital teaching promises to open up courses to learners in diverse locations [8]. 

The German Medical Informatics Initiative (MII) is currently researching and developing methods to share medical data from multiple locations for translational health research [9]. As the acquisition of cross-location and multidisciplinary competencies is an important prerequisite for data sharing, HiGHmed, one of the MII members, is explicitly addressing this within a separate subproject, designed to foster the generation, evaluation and analysis of medical data. For this purpose, HiGHmed is developing the digital teaching organization entitled “HiGHmeducation”, which will facilitate the sustainable operation of a nationwide teaching network. Within HiGHmeducation, individual HiGHmed partners are offering digital teaching modules, which can be accessed by interested parties from any of the partner institutes. 

The teaching modules within HiGHmeducation have been designed both for those interested in studying and for those already in employment. The modules are also open to all disciplines of the health care system, such as medical informatics, medicine, nursing and administration, since the requirements for digital skills are increasing in all occupational groups of the health care system. This interdisciplinary structure should also strengthen interprofessional learning and working. Each module represents a thematically complete course with a defined workload and is based on the didactic framework developed for HiGHmeducation [10]. 

In order to assess whether the didactic methods successfully supported the teaching of each module’s learning objectives, a standardized evaluation is conducted at the end of each module using a German version of the Community of Inquiry questionnaire [11]. The competencies taught in the modules address in particular the current need for increased digital literacy in the training of health care professionals. These competencies are also being addressed by a separate project, which is currently working on the revision of the National Catalogue of Learning Objectives in Medicine (NKLM).

The work of HiGHmeducation, the teaching network, is hampered by various aspects, which are described below; possible solutions to these problems have been offered within other teaching networks; these include the MedizinDidaktikNetz (MDN) as a working group of the Medizinischen Fakultätentag (MFT), the Digitalization Committee of the Gesellschaft für Medizinische Ausbildung (GMA) and the Hochschulforum Digitalisierung (HFD) are active in these areas. For this current paper, this preparatory work by these other organization was utilized as a baseline of knowledge in the planned workshop groups. In particular, the aspects of the German education system, which are hampering HiGHmeducation and were therefore the focus of the planned workshop groups are (in no particular order):

1. Recognition of exam results from other institutions into the curriculum at the home institution.
2. High standards in teaching modules require a considerable investment of both time and effort on the part of the teacher in the development and implementation of the modules. In modern teaching it is important for teachers that this time spent be recognized so that it reflects in their teaching load. For this reason, it should be possible to credit digital teaching in the teaching load appropriately, such that it reflects in the compulsory teaching regulations of the German federal states.
3. The transition from the complementary use of digital technologies in conventional courses to fully digital courses requires new skills on the part of teachers. There are numerous German-language materials for acquiring these skills, making it difficult to provide an overview. Teachers face a challenge in selecting relevant continuing education content.
4. When developing a teaching network and cross-locational modules, scalability for larger numbers of participants must be taken into account from the very beginning. This includes participant interaction and examination practices. For this purpose, technical infrastructure must be developed that effectively supports the process in the environment of German universities.

**Figure 1:**
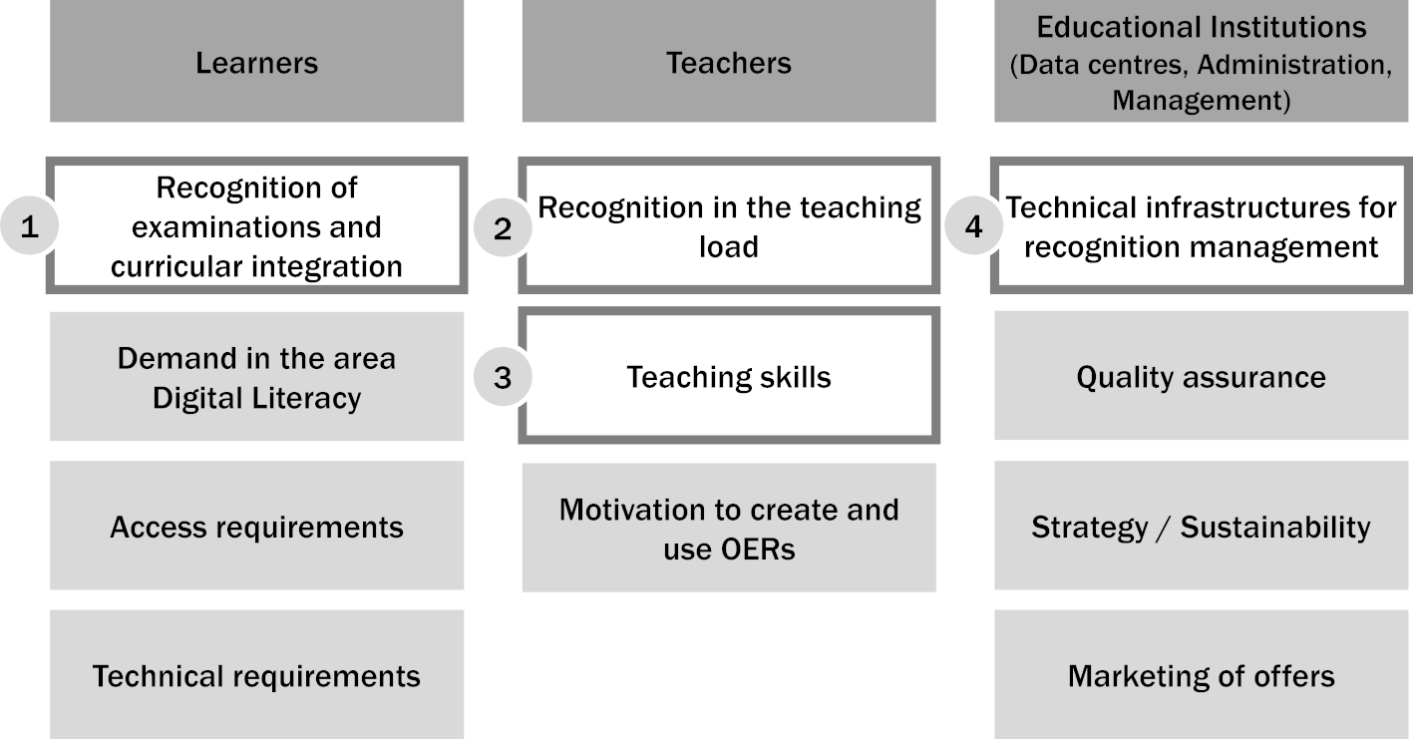
Identified topic groups sorted by primary stakeholders. Topic groups covered at the workshop are highlighted in white and numbered.

These aspects fall within three primary stakeholders “learners”, “teachers” and “educational institutions”. This is shown in figure 1 (Fig. 1).

## Methods

Due to the overarching relevance of the four identified problem areas, a workshop was organized, comprised of experts from three organizations – firstly, the work group “Technology-based Teaching and Learning in Medicine” from the German Society for Medical Informatics, Biometry and Epidemiology (GMDS); secondly, the committee “Digitalization” from the Society for Medical Education (GMA) and thirdly from the HiGHmed consortium. 

Prior to the beginning of the workshop, some representatives from the three organizations discussed their respective experiences with regard to cross-locational teaching. In the process, they identified key problem areas (as outlined above), specifically in the areas of teaching culture, strategic orientation, and organizational and legal structure. This list of problem areas was prioritized according to two factors: firstly, the urgency of the problems from the point of view of the HiGHmeducation consortium and secondly, according to the expertise provided by the aforementioned participating professional societies. The four most relevant topics were selected to be addressed in the workshop. The topics were then sent to the participants before the workshop, so that they could indicate their preferences, based on their experience and expertise. 

At the beginning of the workshop, the participants were divided into groups according to their preferences and the organizations involved. During the workshop, each group began by clarifying and elaborating upon the specifics of the defined problem area, as each area had only been briefly outlined in advance. Each member of these groups contributed their experiences of both the scope of the problem and potential solutions. The solutions from these discussions were then collected and amalgamated into the results; these are presented sequentially below.

### 1. Recognition of exam results and integration into existing curricula

#### Introduction

The teaching experience of the experts present at the workshop shows that the recognition of exam results from other universities is an important prerequisite for increasing participation in external modules. This is due both to the fact that the marks are presented with their certificates or diplomas and also the fact that there is less risk of delaying the completion of degrees. The difficulty lies in crossing the institutional boundary – students need to have their participation in external modules recognized at the institution in which they are enrolled.

##### Questions

1. What functioning mechanisms already exist for the recognition of exams taken at other universities? How can examination achievements be transferred?
2. Should there be restrictions on the proportion of digital forms of teaching?

##### Results 

In principle, recognition can be granted by decisions on a case-by-case basis or by framework agreements. The former represents a precise, but also complex and time-consuming procedure in which each student submits an individual application for recognition of an examination to the respective home institution. The European Credit Transfer System (ECTS) is a helpful instrument for this procedure: in the grading scale recommended since 2009, the distribution of the grades of other students in the same cohort is provided as additional information to the grade in the local grading system [12]. 

In order to avoid duplicating workload on case-by-case decision-making, inter-institutional framework agreements should be sought. One example of a framework agreement is the EU-funded Erasmus program. The basis for recognition between two universities here is named the Learning Agreement [13]. Another example, with regard to the transfer of grades, comes from the University of Würzburg; there, they have developed a solution with concrete mechanisms for converting grades between different systems [14]. This solution is based on the Bavarian formula, which compares the best mark, the lowest pass mark and the mark achieved [15].

Another factor that can affect exam recognition is the coursework format – differences between the proportion of digital formats permitted at the home institution and at external module providers can prevent the recognition of exam results. Such restrictions on the proportion of permitted digital coursework are often stipulated in higher-level legal structures, such as examination regulations and state higher education laws. These restrictions unfortunately contradict the principle which is a key goal of teaching called Constructive Alignment; this principle encourages the orientation of the learning format towards learning goals. It is therefore recommended to adapt statutes in such a way that participation in digital teaching modules can be fully recognized.

##### Outcome

The following steps should be taken for the recognition of teaching modules from consortium partners: **In the short term:** Develop a catalogue of the modules offered within the teaching network including which modules are recognized at which institutions. Collecting the grading systems used at all the sites in the network and their lower pass marks (as the other metrics of the Bavarian Formula are standard across Germany). **In the long term: **Create a framework agreement for the recognition of examinations for all institutions within the teaching network, taking into account heterogeneous grading systems where applicable.

Regarding the restrictions of the teaching forms: **In the short term:** Develop tools, including standardized text and standardized decision-making support, for the digital formats permitted in examination regulations depending on learning goals. Baumgartner has made concrete suggestions as to which teaching arrangements should be chosen for which types of learning objectives [16]. **In the long term:** Adaptation of local examination regulations in the teaching network to standardized examination formats.

#### 2. Recognition of digital teaching in teaching load 

##### Introduction

Cross-location teaching raises recognition questions not only on the part of the students, but also on the part of the teachers. The compulsory teaching regulations (LVVOs) of the federal states define the teaching load. The methods used to calculate the teaching load are usually based on conventional forms of teaching, so it is unclear how the time spent on digital teaching formats should be credited to the teaching load.

##### Questions

1. On which legal level have universities so far solved the problem of the recognition of cross-location teaching in the teaching budget?
2. How should the recognition of digital teaching be regulated in the LVVOs?

##### Results 

There are already numerous teaching associations that offer country-specific or thematically concentrated teaching. What all of these associations have in common is that recognition in the teaching budget is regulated by the legal structures at the lecturer’s university. This results in a multitude of isolated solutions that do not result in a uniform concept.

In order to be able to make suggestions for the recognition of digital teaching that can be applied nationwide (question 2), the work group compiled an overview of the current regulations from the LVVOs of the federal states based on an overview conducted by the Bavarian medical faculties [17]. It was found that almost all of the examined LVVOs contained regulations for the recognition of digital teaching. Older LVVOs often mention relativizing calculation methods, for example by defining that only a certain proportion of the teaching budget may consist of digital teaching. Often this proportion was 25%. More recent LVVOs tended not to distinguish between digital and conventional teaching.

##### Outcome

**In the short term: **The current regulations hinder the further development of digital teaching because they place artificial limits on the crediting of digital teaching hours toward the teaching load. The recognition of digital teaching should not deviate in any way from face-to-face teaching and should be in relation to the workload involved for learners and their support. **In the long term:** A nationally harmonized categorization of teaching forms would significantly simplify the crediting of teaching output for conventional and digital teaching forms across state borders. In addition, the work group recommended explicitly promoting the creation of high-quality digital teaching formats by reducing the teaching load, as is already being done at some universities.

#### 3. Which competencies are uniformly required for teachers in the field of e-learning nationwide?

##### Introduction

The acquisition of appropriate skills for an increasingly digitalized healthcare system makes problem- and task-based learning with authentic digital data and systems essential. In addition, teaching across locations requires online collaborative learning both students and teachers. These evolving educational formats and their contents are constantly changing both the demanded profile of a teacher and the role of teachers in general. They need skills not only in organizing and implementing virtual forms of teaching, but also in designing learning processes and digital learning materials [18].

##### Question

Which existing concepts define the necessary competencies for teachers in digital, cross-location teaching programs?

##### Results 

Instructional design models such as the sequential ADDIE model (named for its five steps: Analyze, Design, Development, Implement, and Evaluate) [19] or the agile SAM (Successive Approximation Model) [20] provide a framework for the planning, development and delivery of a course. Due to its greater popularity, the workshop focused on the ADDIE model. 

During the analysis step, teachers should ask themselves what kind of teaching and learning environment they are in. This includes aspects such as the technical infrastructure, financial and content support, the necessary competencies and the target group. The focus should also be on the organization of the curriculum and the possibilities for networking with other institutions. 

Knowledge of teaching-learning strategies and learning methods is important for the design step. Teachers should be able to weigh up strategies [21], [22] of active learning that are suitable for the goals pursued. In addition, they should be able to consider certain cognitive-psychological prerequisites from multimedia or “cognitive load” theory [23]. 

In the development step, teachers should be able to use certain teaching-learning techniques such as social interaction, retrieval practice, distributed learning [24] or “worked examples”. Knowledge of effective feedback is also essential in order to implement and support the new teaching units in a meaningful way. 

For implementation, teachers should know which participants they need to involve in order to create a successful teaching-learning organization. They should also be able to use computer-mediated communication and instruments for “Classroom Orchestration” effectively. 

Finally, teachers should be aware of the possibilities for support through evaluation. This way, feedback can initiate a dialogue between those involved, which will constantly improve and update teaching. However, they should also be aware of the control and legitimation function of evaluations in order to be able to check whether all participants have performed their services to achieve the desired teaching quality.

##### Outcomes

**In the short term: **Further best practice examples of approaches to the use of digital media should be collected centrally in teaching networks. The common European framework of reference for languages, the “DigCompEdu framework” [18], is an important addition. **In the long term:** Communities of Practice (CoP) should be established throughout the teaching associations in order to sustainably exchange knowledge and instruments for digital teaching. For selected issues, CoPs across teaching networks could also facilitate a meaningful exchange.

#### 4. Technical infrastructure for recognition management

##### Introduction

The hitherto practiced recognition of examination achievements between universities is a cumbersome and non-scalable process, i.e. it cannot be carried out with reasonable resources for a large number of participants. The increase in digital teaching formats is expected to lead to a sharp rise in recognition cases. Technical infrastructure is necessary to automate the recognition process and thereby ensure the smooth operation of cross-location teaching. Legal framework conditions such as the basic data protection ordinance must be taken into account.

##### Questions

1. How are participants authenticated and authorized?
2. How is the proof of the achieved exam performance provided?
3. How are students’ results transmitted and how can the recognition process be supported technically?

##### Results

For the authentication and authorization of learners, digital identities should be used if possible, which are usually already available at the home institutions. This way, both an uncomplicated access to the course is ensured and students can maintain a sense of belonging to their home institution. For the proof of examination performance, integrity must especially be ensured: the recognizing institution must be able to verify that an examination has been taken exactly as shown in the certificate. If there is a large number of participants, a high degree of automation is likely required. For the automated transfer and recognition of performance, standards should be used, which are proposed in the outcomes below.

Since complete digitalization of the recognition process is not considered feasible in the short term, a step-by-step implementation of these standards should be sought.

##### Outcome

**In the short term:** By using the national service DFN-AAI (Authentication and Authorization Infrastructure of the German Research Network) [25] users enrolled for external modules can authenticate themselves with the identity of their home organization. The home organization is clearly defined so that the services provided can be transferred later. Standards such as LTI (“Learning Tools Interoperability” for the exchange of teaching content at the level of individual lessons) of the IMS Global Learning Consortium [26] or xAPI (“Experience API” for the exchange of learning activities) of the Advanced Distributed Learning (ADL) [https://xapi.com] initiative can be used to digitalize and transmit the performance certificates. The support and use of digital certificates of achievement using existing standards such as OpenBadges is helpful to document exam performance [https://openbadges.org/get-started/]. The digitalization of these conventional processes should be the goal for the near future. 

**In the long-term:** Digitalization of paper-based processes is a pragmatic but not sustainable solution. New processes should be defined and introduced that make the best possible use of the existing optimization potential through digitalization. At the MIT Media Lab, for example, so-called BlockCerts were designed on the basis of the Blockchain technology, which represent an open standard for creating, issuing, displaying and verifying certificates [27]. The Blockchain technology is a decentralized architecture for the forgery-resistant storage of information about values (e.g. exam results), which enables an easy exchange between organizations [28]. The implementation of such a modern recognition process is hardly feasible in the short term, as it requires restructuring the existing infrastructure. Nevertheless, the relevance of the process is demonstrated by its designation as a field of application in the German government's block chain strategy published in 2019 [29].

## Summary

The cooperation of the experts from the professional societies and the consortium resulted in a collection of existing solutions, possibilities for action and open questions in the four problem areas. While the outcomes arising from the workshop were discussed in the context of a purely digital teaching format as utilized in HiGHmeducation, the results can also be applied in blended learning arrangements. As strengthen by our experiences with the COVID-19 pandemic, it has been shown that even in a predominantly digital teaching format, in-person classes are still helpful to ensure commitment, practical relevance and a personal learning atmosphere. It is noticeable that all four topics from the workshop presuppose that competence-oriented learning objectives have been defined, which are not only used to develop courses, but also to design legal frameworks and to train teachers.

In addition to the short- and long-term recommendations for action, we see an urgent need for harmonization in the digital representation of exam results in medical informatics. This harmonization, supplemented by syntactic standardization, could offer the possibility to automate the processing of learning objectives received from other institutions. While semantic frameworks in the form of catalogues of learning objectives are developed for specific subjects [30] and are also regularly updated [31], the technical implementation of harmonized specification and exchange formats is an unsolved problem. This makes complex mappings of learning objectives necessary [32] for which supporting tools have been developed [33], but which still require manual work and interpretation. A semantically and syntactically standardized digital recording of learning objectives would not only support the work of curricular integration in teaching networks, but also facilitate the process of implementing subject-specific learning objectives (here in medical informatics), as is already being done in some national catalogs of learning objectives such as the NKLM. Likewise, a newly designed digital recording of learning achievements would considerably simplify the transfer of certified learning achievements across locations.

Based on the results of the workshop, we consider a national action plan and a superordinate institution on the topic of digitalization in university teaching of medical informatics to be a necessary and meaningful intervention to bundle different activities in this area [6]. This institution could be developed from the field of medical informatics following the example of the MFT working group MedizinDidaktikNetz [34]. Possible patrons of such a network could be the Society for Medical Informatics, Biometry and Epidemiology (GMDS) or an organization on the level of the Medical Informatics Initiative (MII). We consider the patronage of such a network to be an important strategic question and plan a further workshop for the next steps, the objective of which is to develop a more precise structure for a network for digital teaching in medical informatics. In this context, cooperation with working groups of the MFT, the GMA, and the HFD will be sought so that generic results can be shared and mutual needs and offers can be coordinated. This may include other thematically related working groups, such as the GMA's Human Resources and Organizational Development Committee on questions of the necessary competencies for teaching staff. The cooperation between the various participants that has begun here provides a basis for the further development of the German educational landscape in the area of cross-institutional cooperation in the field of digitalization in the health care professions. The continuation of the successful cooperation was supported by all participants. Further workshops and joint publications will be organized. The authors are looking forward to external impulses as well as further experts who are interested in active participation.

## Acknowledgements

We thank all participants of the workshop (study group) for their active participation: Marianne Behrends, Christoph Bohne, Barbara Braun, Bas de Leng, Jasmin Decker, Thomas Deserno, Cornelia Fiessler, Peter Heuschmann, Ina Hoffmann, Thomas Koehler, Martin Lemos, Christoph Rensing, Eugenia Rinaldi, Bernd Romeike, Julian Varghese, Joana Warnecke. Special thanks to Alexander Whillier for editing the English article version.

## Funding

This work was supported by the Federal Ministry of Education and Research under grant number 01ZZ1802A.

## Competing interests

The authors declare that they have no competing interests. 

## References

[1] Bischof L, von Stuckrad T (2013). Die digitale (R)evolution? Chancen und Risiken der Digitalisierung akademischer Lehre.

[2] Hochschulforum Digitalisierung (2016). The Digital Turn - Hochschulbildung im Digitalen Zeitalter.

[3] Breitner M, Guhr N (2018). ATLANTIS: Ein nachhaltiger Lehrverbund der Wirtschaftsinformatik.

[4] Müller H (2019). Was die Digitalisierung im Gesundheitswesen erfordert. ÄrzteZ.

[5] Kuhn S, Frankenhauser S, Tolks D (2018). Digitale Lehr- und Lernangebote in der medizinischen Ausbildung. Bundesgesundheitsbl Gesundheitsforsch Gesundheitsschutz.

[6] Haag M, Igel C, Fischer MR, German Medical Education Society (GMA), Committee "Digitization - Technology-Assisted Learning and Teaching", Joint working group "Technology-enhanced Teaching and Learning in Medicine (TeLL)" of the German Association for Medical Informatics, Biometry and Epidemiology (gmds) and the German Informatics Society (GI) (2018). Digital teaching and digital medicine: A national initiative is needed. GMS J Med Educ.

[7] Fisher DH, Fox A (2013). Report on the CCC-CRA Workshop on Multidisciplinary Research For Online Education.

[8] ELAN e. V. (2020). Academic Teaching and LeArning NeTwork in Information Systems.

[9] Knaup P, Deserno T, Prokosch HU, Sax U (2018). Implementation of a National Framework to Promote Health Data Sharing. Yearb Med Inform.

[10] Behrends M, Benning NH, Witte ML, Hoffmann I, Bott OJ (2019). Didactical Framework for Cross-Location Online Learning Modules on Medical Informatics.

[11] Ammenwerth E, Hackl W (2017). Übersetzung und Validierung des Community of Inquiry-Instruments zur Messung erfolgreicher Lernprozesse in online-gestützten Lernsettings.

[12] Generaldirektion Bildung Jugend Sport und Kultur (Europäische Kommission) (2017). ECTS Leitfaden 2015.

[13] European Commission (2019). Erasmus+ Learning Agreement.

[14] Julius-Maximilians-Universität Würzburg (2018). Prüfungs- und Studienordnung für das Zusatzstudium Translational Medicine.

[15] Ständige Konferenz der Kultusminister der Länder in der Bundesrepublik Deutschland (2004). Vereinbarung über die Festsetzung der Gesamtnote bei ausländischen Hochschulzugangszeugnissen.

[16] Baumgartner P, von Beck U, Sommer W, Siepmann F (2008). Blended Learning Arrangement. E-Learning & Wissensmanagement Jahrbuch 2008.

[17] Müller C, Füngerlings S, Tolks D, E-Learning working group in the Competence Network Medical Education in Bavaria (2018). Teaching load - A barrier to digitalisation in higher education? A position paper on the framework surrounding higher education medical teaching in the digital age using bavaria, germany as an example. GMS J Med Educ.

[18] European Commission (2017). European Framework for the Digital Competence of Educators: DigCompEdu.

[19] Branson RK, Rayner GT, Cox LJ, Furman JP, King FJ, Hannum WH (1975). Interservice Procedures for Instructional Systems Development. Executive Summary and Model.

[20] Jung H, Kim YR, Lee H, Shin Y (2019). Advanced instructional design for successive E-learning: Based on the successive approximation model (SAM). Int J E-Learning Corp Gov Heal High Educ.

[21] Dunlosky J, Rawson KA, Marsh EJ, Nathan MJ, Willingham DT (2013). Improving students' learning with effective learning techniques: Promising directions from cognitive and educational psychology. Psychol Sci Public Interes.

[22] Schneider M, Preckel F (2017). Variables associated with achievement in higher education: A systematic review of meta-analyses. Psychol Bull.

[23] Mayer RE, Moreno R (2003). Nine Ways to Reduce Cognitive Load in Multimedia Learning. Educ Psychol.

[24] Carpenter SK, Cepeda NJ, Rohrer D, Kang SHK, Pashler H (2012). Using Spacing to Enhance Diverse Forms of Learning: Review of Recent Research and Implications for Instruction. Educ Psychol Rev.

[25] Verein zur Förderung eines Deutschen Forschungsnetzes e. V. (2019). DFN-AAI - Authentifikations- und Autorisierungs-Infrastruktur.

[26] IMS Global Learning Consortium Inc (2019). IMS Interoperability Standards.

[27] Massachusetts Institute of Technology (2020). Blockcerts - The Open Standard for Blockchain Credentials.

[28] Wright CS (2019). Bitcoin: A Peer-to-Peer Electronic Cash System.

[29] Bundesministerium für Wirtschaft und Energie (2019). Blockchain-Strategie der Bundesregierung.

[30] Dugas M, Röhrig R, Stausberg J (2013). Lernzielkatalog medizinische informatik: Wissen und verantwortung. Dtsch Arztebl Intern.

[31] Varghese J, Röhrig R, Dugas M, GMDS working group “MI-Teaching inMedicine” (2020). Welche Kompetenzen in Medizininformatik benötigen Ärztinnen und Ärzte? Update des Lernzielkatalogs für Studierende der Humanmedizin.GMS Med Inform Biom Epidemiol.

[32] Behrends M, Steffens S, Marschollek M (2017). The implementation of medical informatics in the national competence based catalogue of learning objectives for undergraduate medical education (NKLM). Stud Health Technol Inform.

[33] Fritze O, Lammerding-Koeppel M, Boeker M, Narciss E, Wosnik A, Zipfel S, Griewatz J (2019). Boosting competence-orientation in undergraduate medical education-A web-based tool linking curricular mapping and visual analytics. Med Teach.

[34] Lammerding-Koeppel M, Ebert T, Goerlitz A, Karsten G, Nounla C, Schmidt S, Stosch C, Dieter P (2015). German MedicalTeachingNetwork (MDN) implementing national standards for teacher training. Med Teach.

